# Puberty Suppression for Pediatric Gender Dysphoria and the Child’s Right to an Open Future

**DOI:** 10.1007/s10508-024-02850-4

**Published:** 2024-04-02

**Authors:** Sarah C. J. Jorgensen, Nicole Athéa, Céline Masson

**Affiliations:** 1 Dalla Lana School of Public Health, University of Toronto, 155 College Street, Toronto, ON M5T 3M7 Canada; 2 Ottawa Hospital Research Institute, Ottawa, ON Canada; 3 Hôpitaux de Paris, Paris, France; 4 Département de Psychologie, Université de Picardie Jules-Verne, Amiens, France

**Keywords:** Gender dysphoria, Gender incongruence, Gonadotrophin-releasing hormone analogues, Puberty blockers, Pediatrics, Ethics

## Abstract

In this essay, we consider the clinical and ethical implications of puberty blockers for pediatric gender dysphoria through the lens of “the child’s right to an open future,” which refers to rights that children do not have the capacity to exercise as minors, but that must be protected, so they can exercise them in the future as autonomous adults. We contrast the open future principle with the beliefs underpinning the gender affirming care model and discuss implications for consent. We evaluate claims that puberty blockers are reversible, discuss the scientific uncertainty about long-term benefits and harms, summarize international developments, and examine how suicide has been used to frame puberty suppression as a medically necessary, lifesaving treatment. In discussing these issues, we include relevant empirical evidence and raise questions for clinicians and researchers. We conclude that treatment pathways that delay decisions about medical transition until the child has had the chance to grow and mature into an autonomous adulthood would be most consistent with the open future principle.

## Introduction

One of the most ethically fraught issues in the care of children with gender dysphoria is the use of gonadotrophin-releasing hormone (GnRH) analogues, commonly referred to as puberty blockers. Drugs in this class act on GnRH receptors in the pituitary gland to inhibit the production and release of sex hormones, thereby halting pubertal development (Kumar & Sharma, 2014). Proponents of puberty blockers consider them to be safe and fully reversible (de Vries & Cohen-Kettenis, 2012; Gooren & Delemarre-van de Waal, 1996; HHS, 2022; Rafferty et al., 2018). They point to the potential distress associated with the development of secondary sex characteristics that are incongruent with gender identity and the high rates of self-harm and suicidal ideation reported among children with gender dysphoria (de Snoo-Trimp et al., 2022; de Vries et al., 2021; Giordano, 2008; Kreukels & Cohen-Kettenis, 2011; Rafferty et al., 2018). Additionally, they believe the quality of the evidence supporting puberty blockers has been misrepresented (Lepore et al., 2022; McNamara et al., 2022, 2023), and maintain that respect for the child’s autonomy should generally override concerns about decision-making competence (Ashley, 2022, 2023; Priest, 2019). On the other hand, there is growing recognition that the evidence supporting puberty blockers is very low quality and at high risk of bias, long-term harms and benefits are uncertain (Block, 2023a; Cass, 2022; COHERE, 2020; Ludvigsson et al., 2023; NICE, 2020; Socialstyrelsen, 2022), and the goals of treatment are unclear (Levine & Abbruzzese, 2023). Moreover, potential consequences, which extend beyond physical and psychological outcomes to encompass family and intimate relationships, may be difficult for children to understand and appreciate (Levine, 2018, 2019).

In this essay, we expand upon the debate by considering the clinical and ethical implications of puberty blockers for pediatric gender dysphoria through the lens of “the child’s right to an open future” (Feinberg, 1980, p. 76). The open future principle was introduced by American legal and political philosopher, Feinberg (1926–2004), in a widely cited essay more than 40 years ago. Feinberg’s (1980) open future principle refers to rights that children do not have the capacity to exercise as minors, but that must be protected so they can exercise them when they reach maturity. These rights function as a constraint to ensure certain options are left open for the child as an autonomous adult. The open future principle has been applied to ideological aspects of child-rearing, particularly those related to education and religion (Mills, 2003; Millum, 2014; Morgan, 2005). It has also gained traction in debates about certain bioethical issues, including genetic reproductive technologies (Davis, 1997; Garrett et al., 2019), surgery in infants with disorders of sex development (Harris & Chan, 2019; Kon, 2015), fertility preservation in children facing gonadotoxic cancer treatment (Cutas & Hens, 2015), and infant circumcision (Darby, 2013).

We begin our analysis by briefly revisiting Feinberg’s original formulation of the open future principle. We then apply the open future principle to puberty suppression in the context of gender affirming care and discuss implications for consent. We evaluate claims that puberty blockers are reversible, discuss the scientific uncertainty about long-term benefits and harms, summarize international developments, and examine how suicide has been used to frame puberty suppression as a medically necessary, lifesaving treatment. In discussing these issues, we include relevant empirical evidence and raise questions for clinicians and researchers. While our focus is puberty blockers, many of our arguments could be applied to other medical interventions for pediatric gender dysphoria, including cross-sex hormones and surgery.

## Terminology

Before proceeding, it is necessary to clarify terminology. Throughout this essay, child refers to a person who is younger than 18 years of age (Lansdown & Vaghri, 2022). We use the term adolescent or adolescence when describing maturational changes occurring during puberty or to be consistent with terms used in cited studies. Drugs in the GnRH analogue class include leuprolide, triptorelin, and histrelin. They are administered by intramuscular or subcutaneous injection every 1–6 months, depending on the formulation; a yearly subcutaneous implant is also available. When used for gender dysphoria, GnRH analogues may be started at Tanner stage 2 of puberty, which occurs as early as 9 years of age (Hembree et al., 2017). The term detransitioner refers to an individual who underwent medical and/or surgical transition, and then discontinued medications and/or sought to reverse their surgery (Cass, 2022). Desistance is a term that is often applied to children whose gender dysphoria resolved before undergoing any medical or surgical interventions (Ristori & Steensma, 2016).

## The Child’s Right to an Open Future

In his discussion of children’s rights, Feinberg (1980) distinguishes among three broad categories of rights that can be held by people as adults or children. First, there are rights that both adults and children possess (e.g., the right bodily integrity). Next, there are rights that can only be exercised by autonomous adults (e.g., the right to vote or the right to free exercise of religion). Third, there are rights held primarily by children, which can be further divided into two types. Dependency rights are related to the present well-being of the child (e.g., the right to be provided with basics such as food, shelter, and protection). By contrast, “rights in trust,” refers to the future autonomy that the child will eventually develop (Feinberg, 1980, p. 76). According to Feinberg (1980), these rights, “are to be saved for the child until he is an adult, but [they] can be violated “in advance,” so to speak, before the child is in a position to exercise them” (p. 77). He goes on to explain that violating these rights:

Guarantees now that when the child is an autonomous adult, certain options will already be closed to him. His right while still a child is to have these future options kept open until he is a fully formed self-determining adult capable of deciding among them (Feinberg, 1980, p. 77).

In his seminal essay, Feinberg (1980) left the full scope of rights in trust deliberately vague. He states, “[i]t is plausible to ascribe to children a right to an open future only in some, not all respects, and the simple formula leaves those respects unspecified” (Feinberg, 1980, p. 77). While some have argued for a maximal interpretation of the right’s scope, which would imply all options that might permissibly be chosen by the autonomous adult be protected (Millum, 2014), a more moderate and pragmatic interpretation, whereby the future adult is able to choose among some “vital” interests that are important in determining one’s life course has been applied by many who cite Feinberg’s work (Darby, 2013; Davis, 1997; Garrett, 2022; Kon, 2015; Wenner & George, 2021). We can imagine interests related to pubertal development as rights with this vital quality.

## The Gender Affirming Care Model

Puberty blockers are given as part of gender affirming care–a model of care which involves various combinations of social transition (e.g., change in name, pronouns, clothing, hairstyle, official documents, etc.), endocrine treatments (e.g., puberty blockers and cross-sex hormones), surgery, and mental health support (Coleman et al., 2022). Gender affirming care is structured around the concept of an innate gender identity, which is understood as a deeply felt sense of maleness, femaleness, or otherwise, and can vary independent of sex (Fausto-Sterling, 2012; Hembree et al., 2017; Rafferty et al., 2018). There is no objective test nor biomarker that can confirm this subjective sense of self. Although proponents of gender affirmation recognize that gender identity development is dynamic and can undergo multiple shifts throughout childhood and into adulthood (Ashley, 2019b; Coleman et al., 2022; de Rooy et al., 2022; Fausto-Sterling, 2012; Hembree et al., 2017), they contend that the role of adults is not to question the child’s gender identity nor explore causative factors for their dysphoria, but instead to affirm their gendered self-image and facilitate achievement of their “gender embodiment goals,” through medical intervention, if desired (Ashley, 2022, 2023; Coleman et al., 2022; Rafferty et al., 2018). Legal scholar Ashley (2023) captured this commitment to respect the child’s present autonomy when Ashley stated,

There is typically nobody who is better positioned to make medical decisions that go to the heart of the patient’s identity than the patient themselves. Rather than taking away trans youth’s medical authority, pediatric transgender healthcare should focus on supporting trans youth’s ability to make the best possible decision in light of their values, commitments, and cares (p. 110).

Under the gender affirming care model, the question faced by clinicians and parents is not whether or when children with gender dysphoria can be considered competent to consent to puberty blockers, at least in practice if not in law, but rather how to appropriately involve them in the decision-making process assuming they are competent (de Snoo-Trimp et al., 2022) or even, as has been argued, deferring to their judgement if they lack full decision-making competence (Ashley, 2023).

The gender affirming care model’s emphasis on immediate self-fulfillment and self-determination overlooks important aspects of child and adolescent development. Adolescence is a period marked by major physical growth and rapid increases in mental capabilities, yet despite these maturational changes, overall rates of death and disability increase dramatically between middle childhood and late adolescence (Dahl, 2001, 2004; Patton & Viner, 2007). This has been described as the “health paradox of adolescence” (Dahl, 2004, p. 3). Increases in morbidity and mortality during adolescence are primarily due to outcomes related to difficulties regulating behavior and emotions, e.g., suicide, physical injuries, risky sexual behavior, and alcohol and drug abuse (Dahl, 2001; Patton & Viner, 2007). Evidence from both animal and human studies suggests difficulties controlling behavior and emotions stem from a biologically driven disjunction between novelty and sensation seeking on one hand, both of which increase dramatically at puberty, and the development of self-regulatory competence, on the other, which does not fully mature until early adulthood (Patton & Viner, 2007; Shulman et al., 2016). Thus, although cognitive capabilities involved in decision-making approximate those of adults by mid adolescence, psychosocial factors, such as peer pressure, impulsivity, and risk-seeking can affect–and often impair–an adolescent’s ability to make appropriate decisions (Steinberg, 2005, 2008; Steinberg & Monahan, 2007).

Feinberg (1980) recognized that the child’s future interests are not necessarily promoted by a policy of, “protecting his budding right to self-determination” (p. 91). A child’s current desires and preferences, or in the present context, “gender embodiments goals,” may sometimes clash with their future interests and therefore, respect for a child’s future autonomy as an adult could require preventing their exercise of free choice now (Feinberg, 1980). However, Feinberg (1980) also conceded that there is no sharp dividing line between a child and an autonomous adult:

Any “mere child” beyond the stage of infancy is only a child in some respects, and already an adult in others. Such dividing lines as the eighteenth or twenty-first birthday are simply approximations (plausible guesses) for the point where all the natural rights-in-trust have become actual [adult] rights” (p. 95).

Moreover, he acknowledged that the child plays an “ever-greater role in the creation of his own life, until at the arbitrarily fixed point of full maturity or adulthood, he is at last fully and properly in charge of himself (Feinberg, 1980, p. 96).

Although the gender affirming care model endorses following the lead of the child, studies from the Netherlands have demonstrated that many clinicians at gender clinics, as well as parents of children with gender dysphoria, wrestle with uncertainty when assessing a child’s decision-making competence to consent to puberty blockers (de Snoo-Trimp et al., 2022; Vrouenraets et al., 2015, 2023). It is generally understood that a person needs to fulfill four key criteria to have medical decision-making competence: (1) understand relevant information; (2) appreciate the situation and its consequences; (3) reason about benefits and risks of treatment options; and (4) communicate a choice (Appelbaum & Grisso, 1988), although in practice, competence is typically assessed implicitly in an unstructured manner (Hein et al., 2014; Vrouenraets et al., 2023). Clinicians and parents interviewed by Dutch researchers expressed doubt about whether a child below a certain age could truly understand and appreciate potential long-term health and psychosocial consequences of puberty suppression (de Snoo-Trimp et al., 2022; Vrouenraets et al., 2023). As stated by one clinician in a study focus group,

When do we consider a child as competent for decision-making? Because, […] to oversee the consequences, yes okay, but which consequences? The consequences for a year? For five years? Or for the future moment that they want a child? I feel a lot of struggle here. That I think, yes competent for the consequences in the upcoming period, but not when looking at the consequences for the really long term. But well, […] would you actually know those for any life decision? (de Snoo-Trimp et al., 2022, p. 5).

And to cite another:

That’s what I find difficult about medical decision-making competence: you verify whether someone has understood the information and to what extent someone can appreciate the consequences of the treatment in the future, but to what extent can an 11 year-old understand and appreciate that future properly? I think most 11 year-olds aren’t quite able to do that yet (Vrouenraets et al., 2023, p. 9)

Uncertainty about a child’s capacity to understand the potential impacts of puberty blockers on future fertility was a prominent theme in several studies (de Snoo-Trimp et al., 2022; Vrouenraets et al., 2023). As stated by one clinician,

That [possible infertility due to treatment with puberty suppression and subsequent hormones and surgery] is a very complicated one. As if children of that age [12 or 13 years old] can even begin to imagine what it [infertility] really implies (Vrouenraets et al., 2023, p. 6).

Clinicians and parents were also troubled by the possibility of foreclosing fertility options knowing a child’s desire to have biological children could change as they mature (de Snoo-Trimp et al., 2022; Vrouenraets et al., 2023). This concern was illustrated by one clinician who remarked,

The thing that I feel the most with decision-making competence is: how can you actually know this at this moment? Because we know especially about fertility wishes that this can completely change, even separate from decision-making competence, it can even change between the ages of 30 and 35 (de Snoo-Trimp et al., 2022, p. 6).

A participant who discontinued puberty suppression in her late teens described the evolution of her desire to have children and questioned the decision-making competence of her younger self:

At the moment I know that I would like to have children when I grow older [while at the time I made the decision regarding starting treatment with puberty suppression, I did not have a desire to have children] [...] That’s the only thing I wonder about, whether I was able enough to make that decision at the time (Vrouenraets et al., 2023, p. 6).

Thus, even gender affirming clinicians and parents find assessments of decision-making competence in children with gender dysphoria challenging. While in practice they may assume competence, they clearly wrestle with the knowledge that they may be allowing the child to foreclose future options and transgender individuals themselves retrospectively question their insight and decision-making competence.

## Reversibility of Puberty Blockers

A key rationale for puberty suppression in children with gender dysphoria is the recognition that physical changes associated with endogenous puberty are difficult to reverse, with transgender adults often undergoing expensive and invasive procedures to alter the sex characteristics they developed during puberty (Delemarre-van de Waal & Cohen-Kettenis, 2006; Wenner & George, 2021). Hormonal therapy and surgery in adulthood frequently fails to deliver desired cosmetic changes, especially for male patients, who have been described as having a “never disappearing masculine appearance” (Delemarre-van de Waal & Cohen-Kettenis, 2006, p. S132). It has also been suggested that living in the eschewed gender role during the formative years could contribute to the high rates the mental illness seen in transgender adults following medical and surgical transition (Cohen-Kettenis & Gooren, 1999; Delemarre-van de Waal & Cohen-Kettenis, 2006). As one commentator remarked, “[a]llowing puberty to progress unimpeded thus represents a partially irrovocable decision” (Wenner & George, 2021, p. 929). In the context of the open future principle, not halting endogenous puberty could foreclose a child’s right to achieve their “gender embodiment goals.”

This framing rests on the assumptions that (1) puberty blockers are a low risk, reversible intervention, and (2) that suppressing puberty will improve physical and psychological outcomes in later life. The scientists who first introduced GnRH analogues as a proposed treatment for pediatric gender dysphoria claimed that they were “fully reversible” and that “no lasting undesirable effects are to be expected” (Gooren & Delemarre-van de Waal, 1996, p. 72), although no evidence was cited to support these statements. GnRH analogues have since been promoted as a simple pause to buy children time to explore their gender identity and decide whether to proceed with cross-sex hormones and surgery, without the distress of developing secondary sex characteristics (Cohen-Kettenis et al., 2008; Hembree et al., 2017; Rafferty et al., 2018).

While halting puberty for a short time (i.e., several months) might be expected to have a negligible impact on a child’s development (Biggs, 2023), many children remain on puberty blockers for years (Brik et al., 2020; Carmichael et al., 2021; de Vries et al., 2011; Elkadi et al., 2023), and the reversibility of puberty blockers in this setting has never been proven. Moreover, in practice, puberty suppression is not a distinct, time-limited intervention, but instead the first step in a series of increasingly invasive medical interventions. Multiple studies of children referred to gender clinics have demonstrated that nearly all children who started puberty blockers proceeded to cross-sex hormones, and in time, some will also pursue surgery (Brik et al., 2020; Carmichael et al., 2021; de Vries et al., 2011; Wiepjes et al., 2018). The term gender affirmation “journey” has frequently been used to describe this cascading process (Castagnaro, 2023; Krebs et al., 2022; Weinzimer, 2020).

In medicine, the term “cascade” refers to a process whereby an initiating factor is followed by a stepwise series of events that proceed with increasing momentum to a seemingly inevitable conclusion (Mold & Stein, 1986). Cascades are ubiquitous in multiple areas of medicine and frequently result in iatrogenic harm (Ganguli et al., 2019; Mold & Stein, 1986). They are often inappropriately triggered by factors such as an incomplete patient evaluation, underestimation of treatment risks, or bias toward action (Hoffmann & Del Mar, 2017; Mold & Stein, 1986), and once initiated, they are difficult to stop (Mold & Stein, 1986). For children with gender dysphoria, the initiating factor may have occurred before the child was even referred for clinical assessment, with many children undergoing social transition before referral (Morandini et al., 2022, 2023) and information on medical interventions widely available in schools and through traditional and digital media. In the UK and Australia, increased media coverage of transgender-related topics has been associated with increased referrals to pediatric gender clinics and patients have identified media as a catalyst for them to seek medical care (Pang et al., 2020). Clinicians have also reported that many children appear to have made up their mind about pursuing further medical interventions at the time of starting puberty blockers (Vrouenraets et al., 2022). In a qualitative interview study at two gender clinics in the Netherlands, all children who proceeded with further medical interventions stated that they did not consider puberty suppression to be a way to buy time to explore and decide about further steps; they were already certain that they wanted cross-sex hormones and, for some, surgery when starting puberty blockers (Vrouenraets et al., 2022). While this could be due to a stringent assessment process, most clinicians interviewed for the study stated that some children “only had one goal in mind,” i.e., obtaining hormones and surgery (Vrouenraets et al., 2022, p. 435). One clinician likened the situation to train already on course to its destination: “It is not as if we put them on some kind of train. Those children and adolescents are already on a train when they first visit the clinic and that makes steering the train more difficult” (Vrouenraets et al., 2022, p. 435).

Others have raised concerns that arresting puberty could alter the course of gender identity development, essentially locking-in gender dysphoria that might have resolved during puberty (Cass, 2022; Griffin et al., 2021; Korte et al., 2008). Before gender affirmation became the dominant model of care, clinicians actively worked with children and their families to lessen gender dysphoria or they adopted a neutral approach of “watchful waiting,” where children had access to psychological support to explore reasons for their dysphoria and were allowed to experiment with their gender expression, without criticism or endorsement from adults (Cohen-Kettenis & Pfäfflin, 2003; Zucker, 2008a, 2008b). During this era, 61–98% of cases of early childhood-onset gender dysphoria/gender identity disorder remitted during puberty, if not before (Ristori & Steensma, 2016). Although critics have claimed that earlier studies documenting high rates of desistance inappropriately included children who were merely gender-nonconforming (Olson, 2016; Temple Newhook et al., 2018), a reanalysis of these data that divided children into two groups: those who met the diagnostic threshold for gender identity disorder and those who did not but where nevertheless dysphoric enough to require referral to the gender clinic, showed that those who truly met the diagnostic threshold still had a desistance rate of 67%, while those who were subthreshold desisted at a rate of 93% (Zucker, 2018). Moreover, although it is often claimed that gender dysphoria persisting into early adolescence is highly likely to persist into adulthood (Cohen-Kettenis et al., 2008; de Vries et al., 2011; Gooren & Delemarre-van de Waal, 1996; Vrouenraets et al., 2015), this claim is based on small numbers of cases treated decades ago (Bradley & Zucker, 1997; Smith et al., 2001) and may not generalize to the current cohort of predominantly adolescent females (Butler et al., 2018; Hutchinson et al., 2020; Kaltiala-Heino et al., 2015, 2018; Zucker, 2019), many of whom have complex psychiatric and neurodevelopmental problems (Becerra-Culqui et al., 2018; de Graaf et al., 2018; Kaltiala-Heino et al., 2015; Masson et al., 2023; Thrower et al., 2020).

The growing awareness of detransitioners who deeply regret their transition and feel they were harmed by those who facilitated it attests to our inability to reliably distinguish between those who will derive net benefit from medical transition and those who will be harmed (Butler & Hutchinson, 2020; Entwistle, 2021; Exposito-Campos, 2021; Gribble et al., 2023; Jorgensen, 2023a, 2023b; Littman, 2021; Vandenbussche, 2022). The overall rates of detransition and regret in people who began medical transition as children are largely unknown; there are no large-scale studies with long-term follow-up on this cohort (Cohn, 2023; MacKinnon et al., 2023). Studies describing low rates of regret (i.e., 1–2%) focused primarily on people who transitioned as mature adults and/or are from an era when people had to undergo rigorous psychological screening to be eligible for hormonal therapy and surgery (Bruce et al., 2023; Dhejne et al., 2014; Wiepjes et al., 2018). Moreover, they frequently used narrow definitions of regret such as the application to have birth sex reinstated as legal sex (Dhejne et al., 2014) or request for surgical reversal, to the extent possible (Narayan et al., 2021). Many studies suffered from high rates of loss to follow-up or participant non-response (Blanchard et al., 1989; Bruce et al., 2023; Lawrence, 2003; Rehman et al., 1999; Wiepjes et al., 2018). In one survey, that recruited detransitioners through outreach to sources with diverse perspectives about gender transition, only 24% of respondents reported informing their gender clinic of their detransition (Littman, 2021). Studies that were conducted in the gender affirmation era suggest that between 10 and 30% of those who began medical transition discontinued it within only a few years (Boyd et al., 2022; Hall et al., 2021; Roberts et al., 2022). While not all people who discontinue treatment feel they were harmed or experience regret (Exposito-Campos, 2021; MacKinnon et al., 2022; Pullen Sansfaçon et al., 2023), the high rates of treatment discontinuation seen in recent studies prompted authors of one study to raise questions about “the phenomenon of overdiagnosis, overtreatment, or iatrogenic harm as found in other medical fields” (Boyd et al., 2022, p. 13). Moreover, it can take 8–10 years for regret to manifest in those who transitioned as adults (Dhejne et al., 2014; Wiepjes et al., 2018), after an initial “honeymoon period” lasting from several months to several years (Nobili et al., 2018), suggesting it may be many years before the full extent of regret is known for people who transitioned as children under the gender affirming care model. Last, regret, which implies personal agency, may not be an appropriate endpoint to evaluate iatrogenic harm for those who lacked decisional competence to consent to puberty suppression due to immaturity or co-existing psychiatric or neurodevelopmental conditions (Jorgensen, 2023b).

## Harms and Benefits of Puberty Blockers

When applying the open future principle to children with gender dysphoria, clinicians and parents must balance potential short- and long-term risks and benefits for all treatment options and consider how each alternative could increase or decrease the range of opportunities that will be available to the child in the future. Proponents of puberty blockers have emphasized that allowing endogenous puberty to proceed in children with gender dysphoria is not a neutral act (Coleman et al., 2022). The development of secondary sex characteristics can be a major source of distress and studies have underlined the risk of self-harm as children with gender dysphoria approach puberty (Aitken et al., 2016; Olson et al., 2015). In one study, adolescents with gender dysphoria felt strongly that there was a need to prevent the trauma associated with secondary sex characteristic development, while at the same time they expressed doubts about the ability to make complex decisions regarding medical treatment at a young age (Vrouenraets et al., 2016). In a second study, transgender women who initiated their medical transition as adults expressed disappointment that they did not have a more “feminine” appearance and believed that, had puberty blockers been available to their younger selves, they would have achieved physical changes closer to their desires (Giovanardi et al., 2019, p. 1235). Nevertheless, many transgender adults had concerns about effects of puberty blockers on health and were troubled by the lack of data on long-term outcomes (Giovanardi et al., 2019). Another study reported that most adults (11/15; 73%) who were denied medical and surgical transition as adolescents found other ways to deal with their gender dysphoria and did not feel any regrets about not undergoing transition (Smith et al., 2001). By contrast, interviews with adolescents revealed that uncertainty about long-term negative effects of puberty suppression would not stop them pursuing treatment, with many believing that the chance for relief from current unhappiness was far more important than possible negative long-term consequences of puberty suppression (Vrouenraets et al., 2016), perhaps reflecting adolescents’ tendency to prioritize immediate rewards even when choosing an immediate reward can mean later loss or negative repercussions (Blakemore & Robbins, 2012; Dahl, 2004).

Uncertainty about the benefits and risks of puberty suppression stems partly from the fact that GnRH analogues have never been evaluated in a randomized controlled trial (RCT) for pediatric gender dysphoria, the gold standard for establishing the safety and efficacy of an intervention. Lack of RCT evidence is not unusual in pediatric medicine, however; RCTs rarely enroll pediatric patients, and many medications lack pediatric-specific labeling (Hoon et al., 2019; Ito, 2017). This does not preclude the use of these medications, nor does it make off-label prescribing inherently unsafe. The balance of risks and benefits for many medications commonly used in children has been well-characterized during years of clinical experience. Treatments that have non-negligible risks of serious harm are typically reserved for life-threatening indications. The situation becomes substantially more complex for children with gender dysphoria contemplating puberty blockers however; in addition to an absence of high-quality evidence to guide treatment decisions, the natural trajectory of gender dysphoria in the current cohort of predominantly adolescent females with complex psychiatric and neurodevelopmental conditions (Becerra-Culqui et al., 2018; de Graaf et al., 2018; Kaltiala-Heino et al., 2015; Masson et al., 2023; Thrower et al., 2020) is uncertain. Moreover, while pediatric clinicians frequently rely on observational evidence in the absence of RCTs, observational studies of puberty blockers for gender dysphoria are characterized by multiple methodological weaknesses, making many of them unsuitable to inform clinical practice. Weaknesses include the absence of control groups, inadequate adjustment for confounding, selection bias, high rates of attrition and loss to follow-up, and use of subjective endpoints, which are sensitive to the placebo effect (Abbruzzese et al., 2023; Clayton, 2023; COHERE, 2020; Ludvigsson et al., 2023; NICE, 2020; Socialstyrelsen, 2022). In many studies harms were either not assessed, not reported, or downplayed (de Vries et al., 2014; NICE, 2020) and long-term follow-up has only been (partially) reported for a small number of cases (Asseler, 2022; Cohen-Kettenis et al., 2011; de Rooy et al., 2022).

Therapeutic endpoints are also in flux. Whereas the primary goals of therapy at one time were to reduce gender-related distress, improve coexisting psychiatric symptoms, and prevent self-harm, some have argued that this framing pathologizes transgender identification and excludes those who are otherwise in good mental health (Ashley, 2022; Wenner & George, 2021). Moreover, the high prevalence of psychiatric co-morbidities in those with gender dysphoria is said to be a consequence of chronic stress produced by stigma and discrimination related to gender non-conformity and not indicative of underlying psychiatric pathology (Chodzen et al., 2019; White Hughto et al., 2015). Diverse gender identities are now seen by many as normal variations in subjective human experience and, with the introduction of the informed consent model (Cavanaugh et al., 2016; Chiang & Bachmann, 2023) and “gender incongruence” in the International Statistical Classification of Diseases and Related Health Problems, 11^th^ Revision (WHO, 2023), distress related to gender may no longer be required to access hormonal therapies and surgery (Coleman et al., 2022). Medical interventions are being pursued as a means of achieving self-realization with descriptors such as “gender-euphoria” and “creative transfiguration” appearing in the literature (Ashley, 2019a, p. 481). The contrast between conventional medical care and transgender medical care was summarized as follows:

In conventional medical care, patients go to healthcare professionals to have their illness diagnosed. While they may have opinions on treatment, their ultimate goal is not to obtain a particular intervention but rather to have the underlying condition and its symptoms treated. A rather different picture arises ...[with]...trans health… [When] [s]omeone whose current or developing body conflicts with their gendered self-image…asks a doctor for puberty blockers, hormone therapy, or surgical care… [they] are typically uninterested in the diagnostic process, instead wanting a specific intervention (Ashley, 2022, p. 133).

Management of pediatric gender dysphoria is said to fall in the “special box” of care (Ashley, 2023, p. 112), where medical interventions are required when no medical condition exists, and conventional goals of medicine do not apply (Ashley, 2022). These descriptions are consistent with a recent study that assessed the self-identified goals of transgender identifying adolescents seeking medical care; most adolescents’ goals of care were simply to obtain hormonal therapy and/or surgery (Roden et al., 2023). Medical intervention has become a goal unto itself.

A risk–benefit assessment for puberty blockers that weighs an amorphous concept like “gender embodiment goals” against potential harms gives rise to multiple questions. Gender identity and the importance of gender to an individual’s sense of self can change over time (Ashley, 2019b; Coleman et al., 2022; de Rooy et al., 2022; Fausto-Sterling, 2012; Hembree et al., 2017); gender embodiment goals may also evolve over the course of an individual’s life. Are future gender embodiments goals of the adult, which may differ from those of the child, included in the set of “rights in trust” that should be safeguarded for the autonomous adult to decide? What normative value can be placed on present gender embodiment goals and how does achieving them weigh against future goals as well as potential harms of puberty blockers which may include lifelong medicalization, altered brain development (Chen et al., 2020), diminished bone strength (Biggs, 2021; Lee, 2023), and the loss of the ability to have biological children (Bangalore et al., 2019; Laidlaw et al., 2018; Rosenthal, 2021; Stolk et al., 2023) or experience sexual pleasure (Bowers, 2022)? Additionally, what are the consequences of not reaching psychosocial developmental milestones with peers when puberty is arrested?

The uncertainty about the long-term harms and benefits of puberty blockers for gender dysphoria (Cass, 2022; COHERE, 2020; Ludvigsson et al., 2023; NICE, 2020; Socialstyrelsen, 2022) makes a holistic assessment of their potential impact on a child’s future autonomy rights challenging. Despite the central importance of brain development to a child’s future life course, there remains limited information on the effects of GnRH analogues on cognitive development and emotional and behavioral regulation in humans. It is well recognized that brain remodeling and maturation of executive function are not complete until the mid-twenties (Casey et al., 2008; Steinberg, 2005, 2009). Adolescence is a sensitive period for neural pruning and reorganization of neural circuits, particularly in the frontal cortical regions which control decision-making, judgement, and emotional regulation (Chen et al., 2020; Steinberg, 2005) and hormonal surges during puberty are known to have wide ranging effects on brain structure, function, and connectivity (Blakemore et al., 2010; Chen et al., 2020).

A single case report described a 10-point drop in global IQ and an overall decrease in intellectual performance, “pointing to immaturity in…cognitive development,” in a 12-year-old child 28 months after starting a GnRH analogue for gender dysphoria (Schneider et al., 2017). While it is difficult to draw conclusions from one case, similar findings have been reported in children treated with GnRH analogues for precocious puberty (Mul et al., 2001; Wojniusz et al., 2016). Experiments in animals raise additional questions about the reversibility of effects on cognition. In sheep, peripubertal administration of GnRH analogues reduced long-term spatial memory (Hough et al., 2017b), and this effect remained after discontinuing GnRH analogues (Hough et al., 2017a).

The effects of puberty blockers on the risk of osteoporosis and fractures later in life is a major concern. Puberty is a critical period for determining peak adult bone mass with bone mass approximately doubling from the onset of puberty to young adulthood (Xu et al., 2011). Children with gender dysphoria tend to have lower than expected bone mass based on age and sex before beginning treatment with GnRH analogues, which could be related to decreased physical activity and a high prevalence of eating disorders in this population (Lee, 2023; Lee et al., 2020). Compounding this low baseline bone mass, multiple studies have reported that the expected pattern of bone mass accrual during adolescence does not occur when puberty is paused with GnRH analogues (Biggs, 2021; Boogers et al., 2023; Joseph et al., 2019; Klink et al., 2015; Navabi et al., 2021; Schagen et al., 2020; Vlot et al., 2017), and most studies have found catch-up to be incomplete following the administration of cross-sex hormones (Klink et al., 2015; Stoffers et al., 2019; Vlot et al., 2017), with the possible exceptions of high-dose estrogen in males (Boogers et al., 2023) and testosterone in females who were treated with GnRH analogues for an average of < 2 years (Schagen et al., 2020). Moreover, bone geometry contributes substantially to bone strength and is independently associated with fracture risk (Crabtree et al., 2002; Han & Hahn, 2016). The use of GnRH analogues in early puberty has been shown to alter hip geometry (van der Loos et al., 2021). Initiation in mid to late puberty did not appear to have this effect, suggesting there might be a critical window during which puberty suppression could have lasting effects on bone geometry (van der Loos et al., 2021).

Because GnRH analogues halt gamete maturation, children started on these drugs at Tanner stage 2 of puberty who proceed to cross-sex hormones, as almost all do, will not be able to have biological offspring (Bangalore et al., 2019; Laidlaw et al., 2018; Rosenthal, 2021; Stolk et al., 2023), thus foreclosing what, for many, is their most important and defining life decision. Clinical experience also suggests that they might have impaired or absent sexual function as adults (Bowers, 2022). Delaying or temporarily discontinuing GnRH analogues to promote gamete maturation has been proposed to facilitate fertility preservation (Hembree et al., 2017). However, the procedures involved in fertility preservation are invasive, especially when considering the age of the patient, and the future ability to attain a viable pregnancy and live birth is uncertain (Slonim et al., 2023). In the USA, where cost and inadequate insurance coverage can be additional barriers, < 5% of adolescents receiving GnRH analogues or cross-sex hormones attempted oocyte or semen cryopreservation (Mayhew & Gomez-Lobo, 2020; Nahata et al., 2017). By contrast, 49–67% of transgender identifying adolescents reported a future desire for children (Stolk et al., 2023). Many also acknowledged that their desire for children could change in the future (Stolk et al., 2023). In transgender adults, the desire for children was even higher (54–82%) (Asseler, 2022; Stolk et al., 2023), although only a minority had a stated preference for biological offspring (Stolk et al., 2023).

## International Developments

There are many examples in medicine of novel interventions that were accepted into practice based on low quality evidence that were later found to provide no benefit and some caused significant harm (e.g., prone positioning for sleeping infants (American Academy of Pediatrics, 1996), delayed introduction of peanuts for infants at risk of peanut allergy (Du Toit et al., 2015), routine use of acid blockers for infants with gastroesophageal reflux (Lightdale et al., 2013)). Key lessons from these examples are that greater caution should be exercised in the face of limited evidence and even seemingly well-established practices may still need to be subject to periodic reevaluation (Prasad & Cifu, 2011). Health authorities in several European countries appear to have learned from past mistakes; after conducting systematic reviews of the evidence on puberty blockers for gender dysphoria and finding it to be of very low quality, at high risk of bias, and insufficient to support treatment recommendations, they are restricting these drugs to formal research protocols or “exceptional” cases and instead prioritizing psychological support and treatment of coexisting psychiatric and neurodevelopmental problems (COHERE, 2020; Ludvigsson et al., 2023; NHS, 2023; NICE, 2020; Socialstyrelsen, 2022). An independent systematic review commissioned by England’s National Health Service (NHS) in 2020 concluded that the evidence “suggest[s] little change with GnRH analogues from baseline to follow-up” for outcomes such as gender dysphoria, mental health, and quality of life (NICE, 2020, p. 13) They also noted that there was very limited research on sexual, cognitive, or broader developmental outcomes (NICE, 2020). Sweden’s National Board of Health and Welfare, which sets guidelines for care, determined the risks of GnRH analogues “currently outweigh the possible benefits” (Socialstyrelsen, 2022, p.3). Likewise, the Finnish Health Authority have designated puberty blockers for gender dysphoria an experimental practice and concluded that an improvement in psychiatric symptoms has not been demonstrated with hormonal interventions (COHERE, 2020).

Health authorities in other countries have also urged caution. The French Académie Nationale de Médecine (2022) recently issued a statement emphasizing, “great medical caution must be taken in children and adolescents, given the vulnerability, particularly psychological, of this population and the many undesirable effects, and even serious complications, that some of the available therapies can cause.” Although puberty blockers will still be available, the Académie emphasized, “the greatest reserve is required in their use, given side effects such as impact on growth, bone fragility, risk of sterility, emotional and intellectual consequences and, for girls, symptoms reminiscent of menopause” (Académie Nationale de Médecine, 2022). Norway’s Directorate of Health is in discussions with health services over possible changes to their guidelines for pediatric gender dysphoria after the Norwegian Healthcare Investigation Board (Ukom) found that the evidence for long-term effects of puberty blockers and cross-sex hormones was insufficient (Block, 2023b; Ukom, 2023). In Denmark, puberty blockers and cross-sex hormones are no longer routinely offered to children with gender dysphoria, and instead, therapeutic counselling and support are prioritized (Hansen et al., 2023).

Against this backdrop, in 2022 the United States Department of Health and Human Services (HHS) issued a fact sheet titled, “Gender Affirming Care and Young People,” in which they stated puberty blockers and cross-sex hormones are “crucial to the overall health and well-being” of children suffering from gender dysphoria (HHS, 2022, p. 1). Moreover, they claimed that these interventions are backed by research: “medical and psychosocial gender affirming healthcare practices have been demonstrated to yield lower rates of adverse mental health outcomes, build self-esteem, and improve overall quality of life for transgender and gender diverse youth” (HHS, 2022, p. 1). The American Academy of Pediatrics (AAP) recently reaffirmed its 2018 gender affirming care policy, which includes recommendations supporting the use of puberty blockers in children with gender dysphoria (Rafferty et al., 2018; Wyckoff, 2023). Although the AAP have also commissioned a systematic review of the evidence to inform an expanded set of guidance, Executive Vice President Mark Del Monte, J.D., emphasized the policy authors and AAP leadership, “are confident the principles presented in the original policy….remain in the best interest of children” (Wyckoff, 2023). In a similar departure from the direction of European countries, the World Professional Association for Transgender Health, which provides guidelines for clinicians and advocacy for transgender identifying people, recently released updated “standards of care” in which age thresholds for cross-sex hormones and surgeries have been removed (Coleman et al., 2022).

## Suicide

In the USA and other countries that continue to endorse the gender affirming care model, puberty blockers have been presented as a “tragic compromise,” necessitated by the intractable distress associated with experiencing the “wrong” puberty, together with the risk of suicide if access to these drugs is denied or delayed (Wenner & George, 2021, p. 926). Alarming statistics about suicidal ideation and suicide attempts from online surveys that used convenience sampling, a methodology that produces estimates that lack generalizability (Bornstein et al., 2013), have often been conflated with completed suicides (Biggs, 2022; Levine et al., 2022; Sapir, 2023) and uncritically presented to the public through the media and by public health officials. While evidence suggests that children with gender dysphoria do have higher rates of suicidal ideation and suicide attempts compared with population-matched controls (de Graaf et al., 2022), thankfully, the absolute rate of suicide in this population is still extremely low (Biggs, 2022; Ruuska et al., 2024). A recent nationwide cohort study in Finland found that the suicide rate among young people referred to specialized gender services between 1996 and 2019 was 0.51 per 1000 person years after a mean of 6.5 years follow-up (Ruuska et al., 2024). Likewise, a study using data from England’s Gender Identity Development Service, the world’s largest pediatric gender clinic, found the annual suicide rate was 13 per 100,000 (95% confidence interval 4–34) (Biggs, 2022). Although these suicide rates are approximately four to six times higher than in the general population of comparable age and sex (Biggs, 2022; Ruuska et al., 2024), and the death of a child due to suicide is an unspeakable tragedy, they nevertheless call into question the “tragic compromise” framing. Moreover, as emphasized in a recent commentary, factors that drive an individual to take their own life are incredibly complex and suicide is typically not predictable (Large & Nielssen, 2012). Co-existing psychiatric and neurodevelopmental conditions, as well as adverse childhood experiences, which are frequently overlooked or subsumed when problems are seen through the lens of gender affirmation (Cass, 2022), may contribute substantially to the elevated rate of suicide in children with gender dysphoria (Biggs, 2022; Ruuska et al., 2024). Importantly, there is no compelling evidence that puberty blockers reduce the risk of suicide (Biggs, 2022; Ludvigsson et al., 2023; NICE, 2020; Ruuska et al., 2024), and suggesting otherwise may cause the small number of children who truly are at high risk of suicide to forgo evidence-based suicide prevention interventions (Levine et al., 2022).

Despite these considerations, many clinicians continue to believe that children with gender dysphoria will harm themselves without medical intervention, or at least the promise of future intervention (Vrouenraets et al., 2015), and many parents have been told that their only choice is between a “transgender son or dead daughter,” or vice versa (Kirkup, 2020; Levine et al., 2022; Sapir, 2023). Perceived immediate risks of allowing puberty to proceed unimpeded thus often override concerns about protecting “rights in trust.” This narrative continues to be amplified despite long-standing advise never to speculate about the cause or trigger of suicide since it can influence the behavior of other vulnerable people (MAP, 2017; Samaritans, 2020).

## Conclusions

Decisions about the use of puberty blockers in children with gender dysphoria require that we weigh harms and benefits from both present-oriented and long-term perspectives. While puberty suppression may provide short-term relief from anxious anticipation of pubertal development (Cohen-Kettenis et al., 2008; de Vries et al., 2011), other benefits remain unproven and it entails risks of serious long-term harms, closing off vital options that the child would otherwise have as an autonomous adult (Bangalore et al., 2019; Bowers, 2022; Laidlaw et al., 2018; Lee, 2023; Socialstyrelsen, 2022; Stolk et al., 2023). It recasts a normal physiological process as a disease and can trigger a cascade of increasingly invasive medical interventions. Moreover, no one can know with any certainty how a child’s gender self-image and embodiment goals will evolve over time. As Feinberg (1980) stated, a child’s long-term interests, “cannot be established by determining [their] present desires” (p. 78). To believe otherwise ignores decades of empirical evidence in child and adolescent developmental psychology. Treatment pathways that delay decisions about medical transition until the child has had the chance to grow and mature into an autonomous adult would be most consistent with the open future principle.
